# Cellular Responses in Human Dental Pulp Stem Cells Treated with Three Endodontic Materials

**DOI:** 10.1155/2017/8920356

**Published:** 2017-05-24

**Authors:** Alejandro Victoria-Escandell, José Santiago Ibañez-Cabellos, Sergio Bañuls-Sánchez de Cutanda, Ester Berenguer-Pascual, Jesús Beltrán-García, Eva García-López, Federico V. Pallardó, José Luis García-Giménez, Antonio Pallarés-Sabater, Ignacio Zarzosa-López, Manuel Monterde

**Affiliations:** ^1^Department of Endodontics, Faculty of Medicine and Dentistry, Catholic University of Valencia “San Vicente Mártir”, C/Quevedo, 2, 46001 Valencia, Spain; ^2^Center for Biomedical Network Research on Rare Diseases (CIBERER), CIBER-ISCIII, Valencia, Spain; ^3^Department of Physiology, Faculty of Medicine and Dentistry, University of Valencia, Av. Blasco Ibañez 15, 46010 Valencia, Spain; ^4^INCLIVA Health Research Institute, Av. de Menéndez y Pelayo 4, 46010 Valencia, Spain; ^5^Cell Culture Laboratory, Central Unit of Research in Medicine (UCIM), University of Valencia, Av. Blasco Ibañez 15, 46010 Valencia, Spain

## Abstract

Human dental pulp stem cells (HDPSCs) are of special relevance in future regenerative dental therapies. Characterizing cytotoxicity and genotoxicity produced by endodontic materials is required to evaluate the potential for regeneration of injured tissues in future strategies combining regenerative and root canal therapies. This study explores the cytotoxicity and genotoxicity mediated by oxidative stress of three endodontic materials that are widely used on HDPSCs: a mineral trioxide aggregate (MTA-Angelus white), an epoxy resin sealant (AH-Plus cement), and an MTA-based cement sealer (MTA-Fillapex). Cell viability and cell death rate were assessed by flow cytometry. Oxidative stress was measured by OxyBlot. Levels of antioxidant enzymes were evaluated by Western blot. Genotoxicity was studied by quantifying the expression levels of DNA damage sensors such as ATM and RAD53 genes and DNA damage repair sensors such as RAD51 and PARP-1. Results indicate that AH-Plus increased apoptosis, oxidative stress, and genotoxicity markers in HDPSCs. MTA-Fillapex was the most cytotoxic oxidative stress inductor and genotoxic material for HDPSCs at longer times in preincubated cell culture medium, and MTA-Angelus was less cytotoxic and genotoxic than AH-Plus and MTA-Fillapex at all times assayed.

## 1. Introduction

Progress in dentistry is associated with advancements in dental materials and the design of new regenerative therapies. Both are relevant in the design of restorative biocompatible endodontic materials, which should not affect the cells until the repair of injured tissue has started. Regenerative therapy using human dental pulp stem cells (HDPSCs) is currently acquiring interest because of the potential of these cells to differentiate into odontoblasts and osteoblasts [1–3], both of which have the ability to replace injured bone and dentin pulp tissues with healthy tissue and thus restore functionality of the tooth [4]. HDPSCs can migrate to the pulp lesion sites to replace damaged cells and in turn contribute to the healing process [5]. Therefore, it is recommended that materials used during odontological interventions do not interfere in cellular signaling mediated by HDPSCs.

Biocompatibility is one of the most important requirements for endodontic materials. In vitro studies evaluating biocompatibility by means of cytotoxicity analysis of endodontic materials have been a previous focus for research. Endodontic materials can produce oxidative stress [6, 7] contributing to genotoxicity. However, there are few studies on how these materials can damage DNA and the DNA damage signaling response mediated by them [8].

Among the materials used for endodontics, root canal sealers (RCSs) are used as root filling material in classical endodontic therapy. However, RCSs can extrude to the periarticular area through the apical foramen or the lateral and accessory canals. In this way, they can establish direct contact with periapical tissues where they can stimulate an inflammatory reaction [9, 10] and delay the healing process [11]. Even if there is no extrusion, these materials can permit the release of soluble substances [12] that can be toxic to the periapical tissues and affect the local bone metabolism and the wound healing process [13].

AH-Plus, an RCS widely used in endodontics, contains epoxy resins and amines [14, 15] which can mediate cytotoxicity and genotoxicity [16]. MTA-Fillapex is an RCS containing MTA and a synthetic disalicylate resin. It was created in an effort to combine a material with excellent biocompatibility and bioactive potential such as MTA with another material with very good physical properties such as synthetic resins. However, recent research has provided contradictory results for this sealer regarding cytotoxicity and genotoxicity [17–20].

Mineral trioxide aggregate MTA-Angelus is a root repair material composed of calcium silicate-based hydraulic cement, which has been described as biocompatible and has been commonly used in the repair of pulp exposures and root perforations, among other applications [21]. Previous studies have demonstrated that MTA-Angelus is not genotoxic over short periods of time [22]. However, it lacks the appropriate physical properties to be used as an RCS since it does not have adequate fluidity and it is difficult to manipulate and transport inside the conduct [23].

We propose the use of HDPSCs to characterize cytotoxicity and genotoxicity mediated by oxidative stress produced by endodontic materials, because of their special capability to regenerate injured tissues and for their relevance in stem cell-based regenerative therapy. Moreover, HDPSCs can be used in regenerative therapy of pulp tissue by cell transplantation into the root canal or pulp chamber, reinforcing the necessity of evaluating the effect of these materials on HDPSCs.

In this study, we examined cytotoxicity, DNA damage responses (DDR), and oxidative stress produced by three endodontic materials (MTA-Angelus, AH-Plus, and MTA-Fillapex) in HDPSCs. Apoptosis and necrosis were evaluated by flow cytometry, the expression of genes participating in DDR by qRT-PCR, and oxidative stress and antioxidant enzyme levels by Western blot.

## 2. Materials and Methods

### 2.1. Sample Preparation

Each endodontic sealer (MTA-Angelus, AH-Plus, and MTA-Fillapex) was prepared as indicated in the manufacturer's instructions. The composition of each endodontic material is shown in Supplementary Table S1 available online at https://doi.org/10.1155/2017/8920356 (Supplementary Materials). Test samples consisting of preconditioned cell culture medium were prepared according to ISO 10993-12:2007 [24]. Briefly explained, 100 mg of each freshly mixed RCS (AH-Plus and MTA-Fillapex) and 100 mg of MTA-Angelus powder were immersed in 1 mL of serum-free low-glucose DMEM (Biowest, ref: L0060-500), supplemented with antibiotics and fungicides. These samples were incubated for different time periods comprising 24 h, 48 h, 7 days, 15 days, and 30 days in an incubator at 37°C and under hypoxic conditions of 3% O_2_ and 5% CO_2_. The obtained extracts were filtered using 0.40 *μ*m filters and stored until their use. The original samples were considered 100% stock medium. Prior to their use in the experiments, preconditioned media were supplemented with 10% fetal bovine serum. For each experiment performed, 1 : 2 dilutions of the stock medium were used. The pH of each preconditioned medium was measured using a pH meter (Consort C1010, Cleaver Scientific Ltd., Warwickshire, UK). Triplicates of each preconditioned medium were prepared independently in order to perform three independent replicates for each assay.

### 2.2. Cell Culture

HDPSCs were provided by Marya El Alami and Prof. Juan Gambini (Department of Physiology, Medicine and Dentistry School, University of Valencia). HDPSCs were obtained from extracted teeth of healthy subjects after signing an informed consent and getting approval from the ethics committee of the University of Valencia that the study fulfilled the Declaration of Helsinki principles. HDPSCs were characterized by positive mesenchymal pluripotency markers such as STRO1, OCT1, CD133, CD34, and nestin and by a negative signal for CD45, confirming that the cells conserved mesenchymal stem cell properties [25]. HDPSCs were grown in low-glucose DMEM (Biowest, ref: L0060-500), supplemented with 10% fetal bovine serum (HyClone, ref: SV30160.03), 100 *μ*g/mL penicillin, 100 *μ*g/mL streptomycin, and 0.25 *μ*g/mL amphotericin in a cell culture incubator at 37°C and under hypoxia conditions of 3% O_2_ and 5% CO_2_. To perform cytotoxicity and genotoxicity experiments, HDPSCs were incubated with mediums prepared with endodontic materials described in the sample preparation section. The group defined as the control was exposed only to supplemented DMEM culture medium.

### 2.3. Cytotoxicity Assay

The cytotoxicity of each endodontic material was assessed using the sulforhodamine B (SRB) assay. The protocol was described previously by Vichai and Kirtikara [26]. Briefly explained, HDPSCs were cultured for 24 h in a 96-well plate. Afterwards, the cells were exposed for 24 additional hours to the 1 : 2 dilution of medium preconditioned with endodontic materials as described in the previous section. Cell viability was calculated based on the measurement of the basic amino acid content using 0.4% SRB in 1% acetic acid with the absorbance measurement at 492 nm, subtracting the background measurement at 620 nm. Each condition was tested by triplicate in three independent samples.

### 2.4. Flow Cytometry

Apoptosis was determined with the Annexin V kit (Immunostep, Salamanca, Spain) following the manufacturer's specifications. 10^6^ cells were resuspended in 100 *μ*L of diluted 1X Annexin V binding buffer (Annexin V Binding Buffer, 10X, 0.1 M HEPES NaOH (pH 7.4), 1.4 M NaCl, and 25 mM CaCl2) and stained with 5 *μ*L Annexin V-FITC and 5 *μ*L propidium iodide (PI) for 15 minutes at room temperature in the dark. After the incubation period, 400 *μ*L of 1X Annexin V binding buffer was added. For each sample, 4000 stained cells were analyzed by flow cytometry using a FACS-Verse cytometer (Becton Dickinson, San Jose, CA, USA) and Infinicyt software (Cytognos, Santa Marta de Tormes, Salamanca, Spain). Each condition was tested in triplicate.

### 2.5. Oxidized Protein Analysis by OxyBlot Technique

To determine protein carbonyl groups, we performed the procedure proposed by Shacter et al. [27]. Briefly explained, 10 *μ*g of proteins was denatured and derivatized using 10 mM DNPH in acid solution. The reaction mixture was neutralized and separated by SDS/PAGE and transferred onto a nitrocellulose membrane.

Finally, the membrane reacted to the anti-DNP antibody as described by the manufacturer of the OxyBlot kit (OxyBlot Protein Oxidation Detection kit, Millipore Inc., Billerica, MA. USA). Western blot and OxyBlot experiments were repeated twice.

### 2.6. Antioxidant Enzyme Expression by Western Blot

MnSOD and catalase protein levels were studied by Western blotting, using 20 *μ*g of total protein extracts obtained after cell lysis, as previously described by us in previous papers [28]. The antibodies used were anti-catalase (Sigma, St. Louis, USA) and anti-MnSOD (Stressgen, Ann Arbor, MI, USA) at a dilution of 1 : 1000 in 1% (*w*/*v*) nonfat dry milk TBS-Tween overnight at 4°C. *β*-actin (1 : 1000, Santa Cruz BioTech, USA) was used as a loading control and secondary antibody, and anti-rabbit IgG (Calbiochem, San Diego, CA, USA) was conjugated to horseradish peroxidase at a dilution of 1 : 2500 in 1% (*w*/*v*) nonfat dry milk for 1 h at room temperature. The detection procedure was performed using Amersham RPN 2106 ECL Western Blotting Detection Reagent (GE Healthcare Bio-Sciences AB, Uppsala, Sweden). Images were captured using a GE Healthcare LAS-4000 system.

### 2.7. Gene Expression Analysis Using the qRT-PCR Method

Total RNA was isolated from cells using the PARIS™ (Protein and RNA Isolation System) Kit (Ambion, Austin, TX, USA). For reverse transcription (RT) reactions, 400 ng of the purified RNA was reverse-transcribed using random hexamers with the High-Capacity cDNA Reverse Transcription Kit (P/N 4322171, Applied Biosystems, Foster City, USA).

The mRNA levels were determined by quantitative real-time PCR analysis using an ABI Prism 7900HT Fast Real-Time PCR System (Applied Biosystems, Foster City, CA, USA). The gene-specific primer pairs and probes of TaqMan Gene Expression Assays (Thermo Fisher)were the following: ATM (Hs01112355_g1, Applied Biosystems), RAD53 or CHEK2 (Hs00200485_m1, Applied Biosystems), RAD51 (Hs00947967_m1, Applied Biosystems), PARP-1 (Hs00242302_m1; Applied Biosystems), and GAPDH (Hs02758991_g1, Applied Biosystems), and were used together with the TaqMan Universal PCR Master Mix (P/N 4304437) and reverse-transcribed sample RNA in 20 *μ*L reaction volumes. PCR conditions were 10 min at 95°C for enzyme activation, followed by 40 two-step cycles (15 s at 95°C; 1 min at 60°C). The levels of GAPDH expression were measured in all samples to normalize differences in RNA input, RNA quality, and reverse transcription efficiency. Each sample was analyzed in triplicate, and relative expression was calculated according to the 2^−ΔΔCt^ method [29].

### 2.8. Statistics

Data from three independent experiments, resulting in nine independent samples, are expressed as the mean ± standard deviation (SD). For experiments with three or more groups, comparisons were made using the one-way analysis of variance (ANOVA) to determine the difference between groups (flow cytometry and gene expression by qRT-PCR). When an interaction effect was found, multiple comparisons using the Student-Newman-Keuls post hoc test were performed. Differences were considered statistically significant for *p*values < 0.05. GraphPad Software v6.0 was used for statistical analysis and graphic representations.

## 3. Results

### 3.1. HDPSC Apoptosis Is Increased in Presence of AH-Plus and MTA-Fillapex

Flow cytometry was used to analyze cell viability and cell death in HDPSCs in the presence of 3 different preconditioned mediums and the control group, as described in the Material and Methods section.

**Figure 1:**
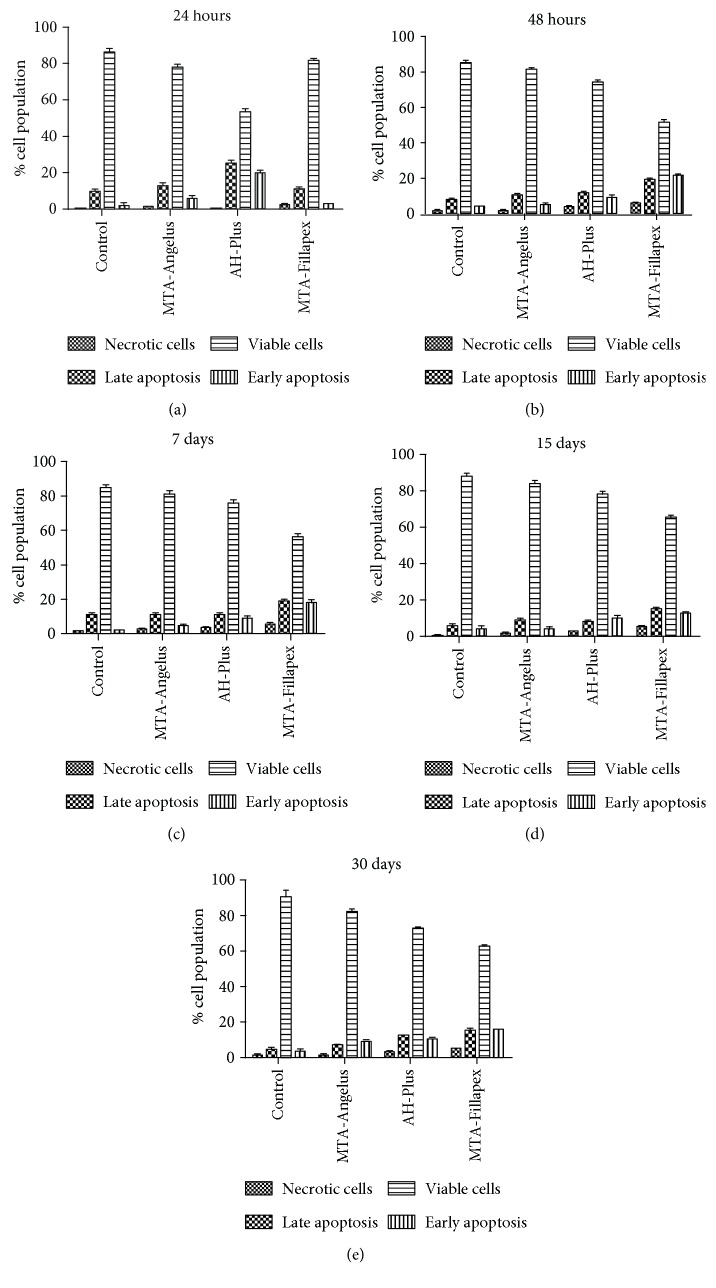
Cytotoxicity induced on HDPSCs assessed using flow cytometry by 1 : 2 dilutions of preconditioned cell culture medium with endodontic materials at 24 hours. Graphs show cell population as viable cells, early apoptotic cells, late apoptotic cells, and necrotic cells at (a) 24 hours, (b) 48 hours, (c) 7 days, (d) 15 days, and (e) 30 days. Each condition was tested by triplicate in three independent samples. The statistical test used was ANOVA with a post hoc Newman-Keuls test to analyze changes in viable, apoptotic, and necrotic cells in each condition. In Table 1, the values of statistical significance for each comparison are shown.

The average of apoptotic cells after incubating samples with preconditioned mediums for 24 hours was significantly different between the 4 groups compared (one-way ANOVA, *p* = 0.007) (Figure 1(a)). As shown in Table 1, when multiple comparisons were performed, the most cytotoxic medium at 24 h was AH-Plus, with significantly increased early apoptosis (ea. 19.9 ± 2.1) and late apoptosis (la. 25.5 ± 1.1) observed, compared to the control group (ea. 2.5 ± 1.2; la. 10.0 ± 1.8), MTA-Angelus (ea. 6.5 ± 0.7; la. 12.9 ± 0.3), and MTA-Fillapex (ea. 3.5 ± 0.2; la. 11.6 ± 0.7).

For longer periods of treatment (48 h and 7, 15, and 30 days), cytometry results revealed that MTA-Fillapex became the most cytotoxic preconditioned medium with higher averages of apoptotic cells (Table 1) and with statistically significant differences in comparison to the other conditions assessed (Figures 1(b), 1(c), 1(d), and 1(e)).

For all conditions assayed, MTA-Angelus was the least cytotoxic endodontic material and showed the highest cell viability values at all times studied (Table 1).

Cell viability and apoptosis of HDPSCs could be related to changes in the pH of the cell culture medium produced by the endodontic materials. Therefore, the pH values were measured at different times during sample preparation (Table 2). Our results indicated that the most basic pH was obtained for MTA-Angelus (pH 8.6 ± 0.5) and MTA-Fillapex (pH 8.6 ± 0.4) at 24 h. The pH of AH-Plus remained near its physiological pH at all times analyzed (pH 7.8 ± 0.3). Results suggest that cell death was not directly affected by the pH of preconditioned DMEM because AH-Plus had the most similar physiological pH and the highest apoptosis at 24 h. Furthermore, pH values for MTA-Angelus and MTA-Fillapex were similar at all times analyzed; however, apoptosis was higher for MTA-Fillapex than for MTA-Angelus, suggesting that pH was not involved in cell cytotoxicity.

### 3.2. Endodontic Materials Induce Oxidative Stress in HDPSCs

**Figure 2:**
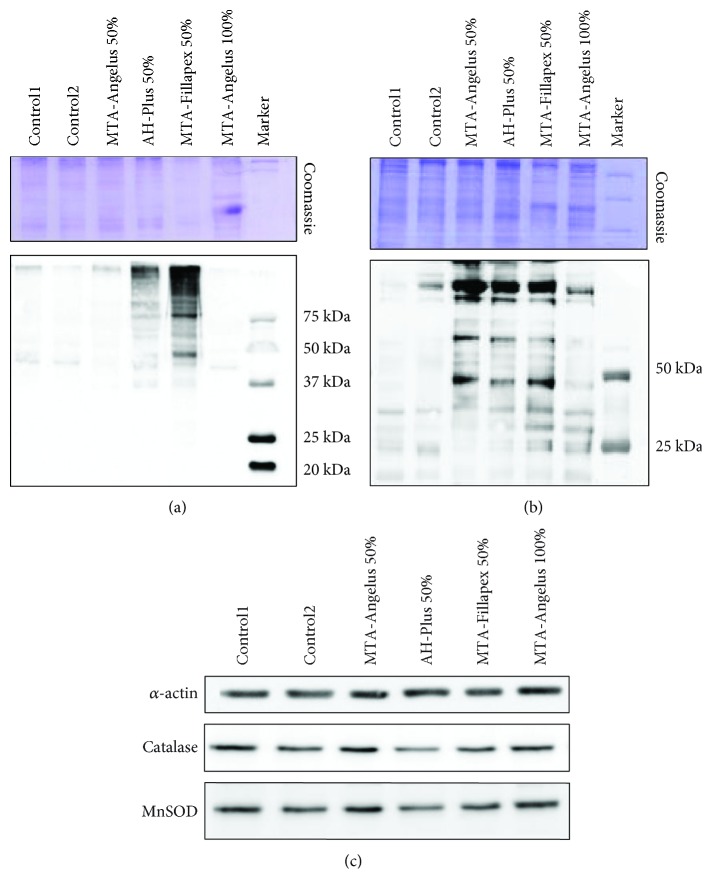
Oxidative stress and antioxidant responses in HDPSCs incubated with endodontic materials. Immunoblots are representative images of two independent analyses in HDPSCs incubated with MTA-Angelus, AH-Plus, and MTA-Fillapex. (a) OxyBlot analysis for the detection of carbonylated proteins from total extracts of HDPSCs incubated with endodontic materials for 24 h. (b) OxyBlot analysis for the detection of carbonylated proteins from total extracts of HDPSCs incubated with endodontic materials for 7 days. (c) Western blot analysis of two antioxidant enzymes, catalase and MnSOD, from total extracts of HDPSCs incubated with endodontic materials for 24 h. For OxyBlots, Coomassie gel staining was used as a loading control. In immunoassays for detecting the levels of antioxidant enzymes, *β*-actin was used as a reference and loading control.

Oxidative stress induced by endodontic materials in HDPSCs was analyzed using the OxyBlot technique. We chose those experimental conditions in which increased apoptosis was observed at 24 h for AH-Plus and MTA-Fillapex. When HDPSCs were incubated in preconditioned medium with endodontic materials AH-Plus and MTA-Fillapex for 24 h, oxidized protein levels increased compared to those in control conditions. Furthermore, a low signal was observed for oxidized proteins for MTA-Angelus, suggesting that this material did not produce oxidative stress at 24 h (Figure 2(a)).

Due to the observed increase in apoptosis in HDPSCs when cells were incubated with MTA-Angelus for 7 days, we also decided to explore oxidative stress in these conditions. We found that MTA-Angelus produced oxidative stress at the same level as AH-Plus and MTA-Fillapex, which may explain the increase in apoptosis observed in these conditions (Figure 2(b)). Furthermore, the concentration of MTA-Angelus in the cell culture media was increased to evaluate the effect of higher concentrations of this endodontic material by observing how oxidized protein levels increased.

All these results suggest that MTA-Angelus was the endodontic material that induced less oxidative stress in HDPSCs.

### 3.3. Endodontic Materials Alter the Expression of Antioxidant Enzymes

Afterwards, we wondered if the oxidative stress induced by endodontic materials in HDPSCs was produced as a consequence of inhibition of key antioxidant enzymes. Using Western blot, we proceeded to evaluate the protein levels of MnSOD and catalase as antioxidant enzymes involved in the detoxification of superoxide radicals and peroxides, respectively (Figure 2(c)). When HDPSCs were incubated in preconditioned medium with the endodontic materials AH-Plus and MTA-Fillapex for 24 h, both catalase and MnSOD were downregulated compared to those in control conditions and MTA-Angelus. Furthermore, we did not observe changes in MnSOD and catalase expression between MTA-Angelus 50% and MTA-Angelus 100%, therefore indicating that this endodontic material did not alter the expression of antioxidant enzymes (Figure 2(c)).

All these results suggest that HDPSCs were under-protected against oxidative stress in the presence of AH-Plus and MTA-Fillapex, while MTA-Angelus did not affect the antioxidant shield in HDPSCs.

### 3.4. Endodontic Materials Affect DNA Damage Responses in HDPSCs

Since the major effects of cytotoxicity analyzed by flow cytometry were found in cell culture medium preincubated for 24 h for AH-Plus and it was also observed that MTA-Fillapex at 48 h of incubation also produced apoptosis, we decided to study the DNA damage responses only at these times.

Among the different types of damage produced in DNA, double-strand breaks (DSBs) and single-strand breaks (SSBs) have mutagenic potential because they can seriously affect the integrity of DNA [30]. DSBs are detected by complex signal transduction mechanisms in which different enzymatic machineries participate, one of the most important being ATM kinase (Ataxia-telangiectasia-mutated protein kinase) [31]. Another component of DNA damage response is the activation of serine/threonine kinase effectors, Rad53 (*Saccharomyces cerevisiae*) being one of the most relevant enzymes or Chk2 (which is its counterpart in humans) [32]. SSBs are detected by PARP-1 [33] which signals the process for repair [34]. In DSB repair, protein Rad51, also known as FANCR, is involved in the guidance of the DNA strands during homologous recombination (HR) [35].

**Figure 3:**
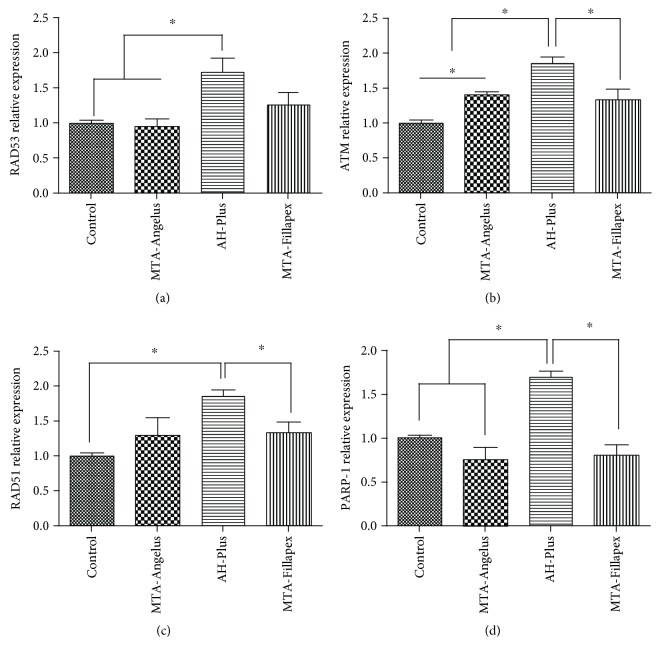
Analysis of expression of DNA damage responses and repair genes in HDPSCs incubated with endodontic materials at 24 hours, by the technique of real-time polymerase chain reaction (qRT-PCR) for (a) RAD53, (b) ATM, (c) RAD51, and (d) PARP-1. The statistical test used was ANOVA with a post hoc Newman-Keuls test to analyze changes in the relative expression of DNA damage response and DNA repair genes. ∗ indicates significant differences between groups compared (*p* < 0.05). Each sample was analyzed in triplicate.

At 24 h, the results show that protein kinase RAD53 (1.7 ± 0.3) (Figure 3(a)) and the ATM kinase (2.0 ± 0.2) (Figure 3(b)) were overexpressed when HDPSCs were incubated with AH-Plus medium compared to the control group. Increased expression of the ATM gene was also observed for the other biomaterials (MTA-Angelus 1.4 ± 0.1 and MTA-Fillapex 1.3 ± 0.3) compared to the control group (1.0 ± 0.1) although the main effect was further increased when the medium preincubated with AH-Plus was used (2.0 ± 0.2). In addition, when the genes participating in DNA repair were analyzed, increased expression of RAD51 was observed when HDPSCs were incubated with AH-Plus (1.9 ± 0.1) and MTA-Fillapex (1.3 ± 0.3) compared to control (1.0 ± 0.1) and MTA-Angelus (1.3 ± 0.4) (Figure 3(c)) and increased expression for PARP-1 was seen when HDPSCs were incubated with AH-Plus (1.8 ± 0.1) compared to other conditions in which relative expression of PARP-1 was similar to the control group (1.0 ± 0.1) (Figure 3(d)) at 24 h. The results suggest that AH-Plus induced both DSBs and SSBs, while MTA-Fillapex only produced the activation of DSB repair.

**Figure 4:**
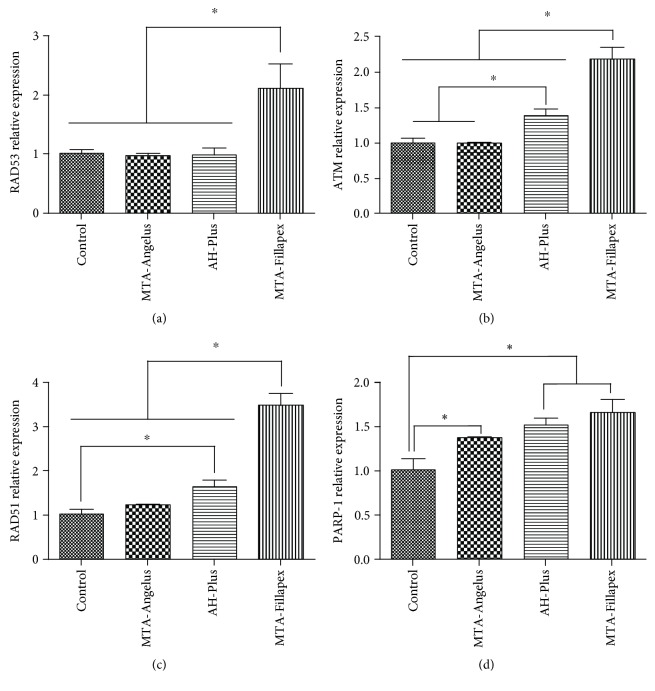
Analysis of expression of DNA damage responses and repair genes in HDPSCs incubated with endodontic materials at 48 hours, by the technique of real-time polymerase chain reaction (qRT-PCR) for (a) RAD53, (b) ATM, (c) RAD51, and (d) PARP-1. The statistical test used was ANOVA with a post hoc Newman-Keuls test to analyze changes in the relative expression of DNA damage response and DNA repair genes. ∗ indicates significant differences between groups compared (*p* < 0.05). Each sample was analyzed in triplicate.

However, at 48 h, when cells were incubated with the medium preconditioned with endodontic materials, increased expression of these genes was observed for cells incubated with MTA-Fillapex (and to a lesser extent for AH-Plus and MTA-Angelus (Figure 4)).

These results indicate that endodontic materials activate DNA repair mechanisms for both DSBs and SSBs. However, the highest activation of DNA damage sensors Rad53 (2.1 ± 0.7) and ATM (1.9 ± 0.5) was found for MTA-Fillapex when results were compared to those of control and other conditions. In line with these results, the highest effector signaling for DNA repair (mediated by Rad51 and PARP-1) was also found for MTA-Fillapex (Rad51 3.5 ± 0.5; PARP-1 1.7 ± 0.3). Relative expression found for these genes in other groups was lower than that found for MTA-Fillapex, control (Rad51 (1.0 ± 0.2), PARP-1 (1.0 ± 0.2)), MTA-Angelus (Rad51 (1.2 ± 0.0), PARP-1 (1.4 ± 0.0)), and AH-Plus (Rad51 (1.5 ± 0.0), PARP-1 (1.4 ± 0.1)). All in all, the results suggest that MTA-Fillapex was the most genotoxic material for HDPSCs.

## 4. Discussion

In our study, we used mesenchymal stem cells from dental pulp (HDPSCs), which have the advantage over other cell lines of being able to differentiate into odontoblasts [1] and osteoblasts [2]. HDPSC is a cell model with physiological properties which are homologous to the primary tissue where the endodontic materials are in contact. Furthermore, HDPSCs have other advantages such as a large capacity for proliferation, the ability to maintain their cellular phenotype for a long time period, and the sensitivity of response to toxins [36, 37]. Additionally, HDPSCs are promising cell lines that can be used in regenerative medicine to repair damaged tissue, and they have potential applicability in dental tissue engineering and regenerative therapy of dental tissues [38]. For these above-mentioned reasons, HDPSCs can be considered a relevant cellular model to evaluate the effect of endodontic materials.

The results obtained by flow cytometry indicated that MTA-Angelus was the least cytotoxic material over time, compared to AH-Plus and MTA-Fillapex. Our results are similar to those obtained by Zhou et al. [39]. These authors studied the effect of MTA in human gingival fibroblasts using flow cytometry and observed that MTA in a diluted medium did not increase apoptosis or necrosis. In contrast, Petrovic et al. [40] found that MTA-Angelus was cytotoxic using 50% diluted medium in an MRC5 cell line consisting of human lung fibroblasts. In our experiments, we observed maximal apoptosis for mediums prepared with AH-Plus at 24 h and MTA-Fillapex at 48 h. Regarding AH-Plus, some studies have shown that this endodontic material increased cytotoxicity at 24 h [41, 42], probably due to the presence of amines in its composition [43]. MTA-Fillapex was the most cytotoxic endodontic material, in agreement with Zhou et al. [44] who observed that MTA-Fillapex was cytotoxic for human gingival fibroblasts using 50% diluted medium preincubated with this material for periods of 1 to 4 weeks.

Oxidative stress can mediate cytotoxicity and genotoxicity, and distinct endodontic materials have demonstrated their ability to generate oxidative stress [6, 45]. Therefore, we were interested in evaluating the oxidative damage and antioxidant defenses in order to assess the oxidative stress in our samples.

The results of our study indicated that MTA-Angelus did not induce oxidative stress in HDPSCs after 24 h of incubation. However, AH-Plus and MTA-Fillapex under these conditions increased oxidative stress in HDPSCs. Coinciding with our results, a study by Camargo et al. [46] evaluated the production of reactive oxygen species (ROS) by white and grey MTA on transfected HDPSCs. The results of ROS production by the cells exposed to the 1 : 1 extracts for 1 hour showed that both white and gray MTA did not cause an increase in ROS production. However, a study by Chang et al. [47] on the ability of MTA-Angelus and other endodontic calcium silicate-based materials to induce the formation of ROS and activate the endogenous antioxidant defenses demonstrated that MTA-Angelus induced production of ROS after 3 days of incubation in an immortalized cell line of human dental pulp. AH-Plus contains bisphenol A diglycidyl ether (BADGE), and some controversial results can be found in the literature suggesting that BADGE can release small amounts of bisphenol A (BPA) [48–51]. However, although no released BPA was the origin of cytotoxicity, it can be considered that BADGE can mediate cytotoxic effects on different cellular models, such as lymphocytes [52] and Caco-2 cells [53].

A study by Kim et al. [54] obtained similar results to those obtained by us, when studying the cytotoxicity of AH-Plus and its ability to produce ROS in an MC-3T3 E1 mouse osteoblast cell line, which was cultured in a medium supplemented with AH-Plus at 30% concentration for 24 hours.

In addition, Camargo et al. [55] analyzed the cytotoxic effect generated by AH-Plus at 50% concentration and mediated by ROS on a fibroblast cell line from human dental pulp.

The mechanisms by which 50% MTA-Fillapex produces oxidative stress and genotoxicity may be mediated by the presence of titanium dioxide (TiO2) in its composition, which has been shown to produce ROS that leads to oxidative damage in DNA [56]. An in vivo study by Zmener et al. [57] evaluated the inflammatory response induced by MTA-Fillapex after subcutaneous implantation of this biomaterial in Wistar rats. The results showed a severe reaction after 10 days that was maintained at 30 days and 90 days. These authors speculate that cytotoxicity may be due to the leaching of toxic elements due to the high solubility of MTA-Fillapex.

Our results showed that in HDPSCs treated with AH-Plus and MTA-Fillapex for 24 h, both catalase and MnSOD were downregulated compared to those in control conditions and MTA-Angelus. Villeneuve et al. have described the fine balance between cell viability and death by controlling ROS levels via Nrf2 and p21 [58]. The authors propose that in mild oxidative stress conditions (such as may occur for MTA-Angelus in our study), cells can respond activating Nrf2 expression and downstream gene targets (such as antioxidant enzymes catalase and MnSOD). However, at high levels of oxidative stress (such as may occur for AH Plus and MTA Fillapex) (Figure 2(c)), one may speculate that the Nrf2 antioxidant response pathway must be suppressed to induce apoptosis, because apoptosis requires the accumulation of ROS (Figures 2(a) and 2(b)). Other plausible explanation is that the Nrf2 system is activated in any case but oxidative stress exceeds the capacities of enzymatic antioxidants, when cells are treated with AH-Plus and MTA-Fillapex.

Therefore, since these materials can produce oxidative stress, it is crucial to evaluate the mechanisms of genotoxicity. In this regard, we studied the expression of different genes mediating cellular response and DNA repair after DNA damage induction. We analyzed sensors and effectors of DNA damage response such as ATM, RAD53, RAD51, and PARP-1.

The cellular medium prepared with AH-Plus for 24 h was the most genotoxic for HDPSCs and produced the overexpression of ATM and RAD53 (Figure 3). AH-Plus also induced the overexpression of RAD51 and PARP-1 in the same conditions. Our results are in agreement with the results we obtained by flow cytometry, confirming a genotoxic effect for AH-Plus. The results for this endodontic material are probably related to the release of formaldehyde during polymerization [59], which is extremely reactive and can cause crosslinks between biomolecules [60]. Our results agree with those obtained by Candeiro et al. in a model of human gingival fibroblasts [61] and also with results obtained by Camargo et al. [16] for this endodontic material. Interestingly, Van Landuyt et al. [62] studied the genotoxicity and cytotoxicity effect mediated by AH-Plus in a model of gingival fibroblasts, in which they did not observe increased levels of gamma-H2AX (a marker for DNA double-strand breaks). However, they found increased cytotoxicity for 1 : 3 and 1 : 10 dilutions of medium preconditioned with AH-Plus. In our study, AH-Plus was able to induce DNA damage and DNA repair activation for both DSBs and SSBs, which were detected by the overexpression of RAD51 and PARP-1, respectively.

When we used medium preincubated with endodontic materials for 48 h, our results demonstrated that MTA-Fillapex further increased the expression of the DNA damage signaling pathways mediated by the ATM and RAD53 sensors and the PARP-1 and RAD51 effectors for DNA repair genes, suggesting that MTA-Fillapex can produce SSBs and DSBs. The results also coincide with the results obtained by flow cytometry and indicate the cytotoxic and genotoxic potential of MTA-Fillapex. The genotoxicity of MTA-Fillapex could be related to the content of TiO2, which has been previously demonstrated to induce the formation of micronuclei [56] and the presence of salicylates in its composition, which were shown to induce DNA damage and apoptosis in vitro in fibrosarcoma cell lines [63]. Bin et al. [64] demonstrated genotoxicity and cytotoxicity for MTA-Fillapex in vitro using V79 fibroblasts and lower concentrations of MTA-Fillapex than those used in our study.

Our results point out the relevance of using an appropriate cell line to study the cytotoxicity, genotoxicity, and biocompatibility of endodontic materials. Particularly, MTA has a wide range of possibilities in endodontic treatments [65] because of its clinical use involving the direct contact of this biomaterial with periradicular and pulpal tissues, contributing not only to cytotoxicity, genotoxicity, and proliferation but also to the differentiation of odontoblasts and osteoblasts [66, 67].

## 5. Conclusions

AH-Plus and MTA-Fillapex were the most cytotoxic and genotoxic materials for HDPSCs in this study. Genotoxicity is mediated by an increase in oxidative stress and downregulation of the antioxidant defense shield. On the other hand, MTA-Angelus was the least cytotoxic and genotoxic material at all assayed times in which antioxidant enzyme expression levels were not altered. This is of special relevance in characterizing the biocompatibility and the cytotoxic and genotoxic effects of the biomaterials on a relevant source of cells for regenerative therapy.

## Supplementary Material

Table S1. Composition of MTA-Angelus White (Angelus, Londrina, PR, Brazil), AH-Plus (Dentsply De Trey, Konstanz, Germany), and MTA-Fillapex (Angelus, Londrina, PR, Brazil).

## Figures and Tables

**Table 1:** Cell viability, apoptosis, and necrosis in HDPSCs at different times of treatment with endodontic materials.

Condition	Group	Viable cells				Early Apoptosis				Necrosis				Late apoptosis			
		Mean	SD	*p*		Mean	SD	*p*		Mean	SD	*p*		Mean	SD	*p*	
Endodontic materials (50%) 24 h	Control	86.8	2.8	—		2.5	1.2	—		0.9	0.1	—		10.0	1.8	—	
	MTA-Angelus	78.5	1.2	C	0.036	6.5	0.7	C	n.s	2.0	0.2	C	0.09	12.9	0.3	C	n.s
				AH-Plus	0.001			AH-Plus	0.002			AH-Plus	0.003			AH-Plus	0.002
				MTA-Fillapex	n.s			MTA-Fillapex	n.s			MTA-Fillapex	n.s			MTA-Fillapex	n.s
	AH-Plus	54.0	0.9	C	<0.0001	19.9	2.1	C	0.001	0.5	0.1	C	n.s	25.5	1.1	C	0.001
				MTA-Angelus	0.001			MTA-Angelus	0.002			MTA-Angelus	0.003			MTA-Angelus	0.002
				MTA-Fillapex	<0.0001			MTA-Fillapex	0.001			MTA-Fillapex	0.001			MTA-Fillapex	0.001
	MTA-Fillapex	82.2	1.1	C	n.s	3.5	0.2	C	n.s	2.7	0.2	C	0.002	11.6	0.7	C	n.s
				MTA-Angelus	n.s			MTA-Angelus	n.s			MTA-Angelus	n.s			MTA-Angelus	n.s
				AH-Plus	<0.0001			AH-Plus	0.001			AH-Plus	0.001			AH-Plus	0.001
Endodontic materials (50%) 48 h	Control	85.9	0.7	—		4.4	0.2	—		1.5	0.9	—		8.1	1.4	—	
	MTA-Angelus	81.8	0.8	C	n.s	5.5	0.6	C	n.s	1.7	0.6	C	n.s	10.9	0.8	C	n.s
				AH-Plus	0.016			AH-Plus	0.012			AH-Plus	n.s			AH-Plus	n.s
				MTA-Fillapex	<0.0001			MTA-Fillapex	<0.0001			MTA-Fillapex	0.018			MTA-Fillapex	0.006
	AH-Plus	74.5	1.6	C	0.03	9.6	1.0	C	0.005	3.9	0.6	C	n.s	11.9	1.2	C	n.s
				MTA-Angelus	0.016			MTA-Angelus	0.012			MTA-Angelus	n.s			MTA-Angelus	n.s
				MTA-Fillapex	<0.0001			MTA-Fillapex	<0.0001			MTA-Fillapex	n.s			MTA-Fillapex	0.01
	MTA-Fillapex	51.9	1.4	C	<0.0001	22.6	0.1	C	<0.0001	6.2	0.8	C	0.015	19.6	0.7	C	0.002
				MTA-Angelus	<0.0001			MTA-Angelus	<0.0001			MTA-Angelus	0.018			MTA-Angelus	0.006
				AH-Plus	<0.0001			AH-Plus	<0.0001			AH-Plus	n.s			AH-Plus	0.01
Endodontic materials (50%) 7 d	Control	85.4	1.5	—		1.8	0.3	—		1.6	0.3	—		11.1	1.4	—	
	MTA-Angelus	81.4	3.1	C	n.s	4.7	1.1	C	n.s	2.7	0.7	C	n.s	11.2	1.2	C	n.s
				AH-Plus	n.s			AH-Plus	n.s			AH-Plus	n.s			AH-Plus	n.s
				MTA-Fillapex	0.005			MTA-Fillapex	0.017			MTA-Fillapex	0.016			MTA-Fillapex	0.012
	AH-Plus	76.1	2.9	C	n.s	8.9	2.6	C	n.s	3.5	0.6	C	n.s	11.3	0.9	C	n.s
				MTA-Angelus	n.s			MTA-Angelus	n.s			MTA-Angelus	n.s			MTA-Angelus	n.s
				MTA-Fillapex	0.013			MTA-Fillapex	n.s			MTA-Fillapex	0.041			MTA-Fillapex	0.013
	MTA-Fillapex	56.5	3.9	C	0.003	17.9	3.3	C	0.009	6.3	0.6	C	0.006	19.2	1.2	C	0.011
				MTA-Angelus	0.005			MTA-Angelus	0.017			MTA-Angelus	0.016			MTA-Angelus	0.012
				AH-Plus	0.013			AH-Plus	n.s			AH-Plus	0.041			AH-Plus	0.013
Endodontic materials (50%) 15 d	Control	88.0	3.8	—		4.6	2.3	—		1.2	0.1	—		6.1	1.4	—	
	MTA-Angelus	84.1	2.8	C	n.s	4.7	0.8	C	n.s	1.9	0.8	C	n.s	9.2	1.2	C	n.s
				AH-Plus	n.s			AH-Plus	n.s			AH-Plus	n.s			AH-Plus	n.s
				MTA-Fillapex	0.009			MTA-Fillapex	0.019			MTA-Fillapex	0.015			MTA-Fillapex	0.027
	AH-Plus	78.5	1.3	C	n.s	10.6	1.5	C	n.s	2.9	0.5	C	n.s	8.3	1.3	C	n.s
				MTA-Angelus	n.s			MTA-Angelus	n.s			MTA-Angelus	n.s			MTA-Angelus	n.s
				MTA-Fillapex	0.033			MTA-Fillapex	n.s			MTA-Fillapex	0.044			MTA-Fillapex	0.018
	MTA-Fillapex	65.4	1.6	C	0.005	13.3	0.1	C	0.018	5.7	0.8	C	0.008	15.6	0.7	C	0.007
				MTA-Angelus	0.009			MTA-Angelus	0.019			MTA-Angelus	0.015			MTA-Angelus	0.027
				AH-Plus	0.033			AH-Plus	n.s			AH-Plus	0.044			AH-Plus	0.018
Endodontic materials (50%) 30 d	Control	88.8	7.8	—		3.4	2.7	—		1.5	0.7	—		4.7	2.3	—	
	MTA-Angelus	82.4	1.9	C	n.s	8.9	1.6	C	n.s	1.8	0.5	C	n.s	693	1.0	C	n.s
				AH-Plus	n.s			AH-Plus	n.s			AH-Plus	n.s			AH-Plus	n.s
				MTA-Fillapex	0.04			MTA-Fillapex	n.s			MTA-Fillapex	0.009			MTA-Fillapex	0.015
	AH-Plus	72.9	0.8	C	0.07	10.6	1.1	C	0.049	3.8	0.4	C	0.042	12.7	0.1	C	0.02
				MTA-Angelus	n.s			MTA-Angelus	n.s			MTA-Angelus	n.s			MTA-Angelus	n.s
				MTA-Fillapex	n.s			MTA-Fillapex	n.s			MTA-Fillapex	n.s			MTA-Fillapex	n.s
	MTA-Fillapex	63.1	1.1	C	0.015	15.9	0.2	C	0.007	5.4	0.3	C	0.007	15.6	1.2	C	0.007
				MTA-Angelus	0.04			MTA-Angelus	n.s			MTA-Angelus	0.009			MTA-Angelus	0.015
				AH-Plus	n.s			AH-Plus	n.s			AH-Plus	n.s			AH-Plus	n.s

**Table 2:** Mean and standard deviations of the pH value for preconditioned medium at the different time periods.

	After DMEM preparation	24 h	48 h	72 h	7 days	15 days	28 days
Control	7.4 ± 0.2	7.4 ± 0.3	7.4 ± 0.2	7.5 ± 0.3	7.6 ± 0.4	7.4 ± 0.3	7.5 ± 0.1
MTA-Angelus	8.7 ± 0.5	8.6 ± 0.5	8.1 ± 0.3	8.0 ± 0.4	7.8 ± 0.2	8.0 ± 0.4	7.9 ± 0.4
AH-Plus	7.6 ± 0.4	7.8 ± 0.3	7.8 ± 0.4	7.6 ± 0.2	7.4 ± 0.3	7.6 ± 0.3	7.5 ± 0.3
MTA-Fillapex	8.8 ± 0.5	8.8 ± 0.4	8.3 ± 0.5	8.0 ± 0.3	8.0 ± 0.2	8.1 ± 0.3	8.1 ± 0.4
