# Supplementary material for: Cellular Responses in Human Dental Pulp Stem Cells Treated with Three Endodontic Materials
Source: Stem Cells Int. 2017 May 24;2017:8920356. doi: 10.1155/2017/8920356 (PMC5511667; doi:10.1155/2017/8920356)
Supplement: Supplementary file 1 — Table S1. Composition of MTA-Angelus White (Angelus, Londrina, PR, Brazil), AH-Plus (Dentsply De Trey, Konstanz, Germany), and MTA-Fillapex (Angelus, Londrina, PR, Brazil). [file 8920356.f1.pdf]

**Table S1.** Composition of MTA-Angelus White (Angelus, Londrina, PR, Brazil), AH-Plus (Dentsply De Trey, Konstanz, Germany), and MTA-Fillapex (Angelus, Londrina, PR, Brazil).

| Endodontic material | Chemical composition | Percentage |
|---|---|---|
| **MTA-Angelus** | Tri-calcium silicate | 51% |
| | Di-calcium silicate | 10% |
| | Tri-calcium aluminate | 11% |
| | Ferroaluminate tri-calcium | 3% |
| | Calcium oxide | 5% |
| | Bismuth oxide | 20% |
| **AH-Plus** | **Chemical composition** | **Percentage** |
| | **Paste A** | |
| | Bisphenol-A epoxy resin | 25-50% |
| | Bisphenol-F epoxy resin | 2.5-10% |
| | Calcium tungstenate<br>Zirconium oxide<br>Sílica<br>Iron oxide | 40-72.5% |
| | **Paste B** | |
| | Dibenzyl-diamine | 10-25% |
| | Aminoadamantane | 2.5-10% |
| | Tri-ciclodecane diamine<br>Calcium tungstenate<br>Zirconium oxide<br>Silica<br>Silicone oil | 65-87.5% |
| **MTA-Fillapex** | **Chemical composition** | **Percentage** |
| | **Paste A** | |
| | Methyl salicylate butylene glicol colophony | 44% |
| | Bismuth trioxide | 35% |
| | Fumed silicon dioxide | 5% |
| | Titanium dioxide | 16% |
| | **Paste B** | |
| | MTA | 44% |
| | Pentaerythritol rosinate | 47% |
| | Fumed silicon dioxide | 6% |
| | Titanium dioxide | 3% |
